# Real‐world treatment patterns and clinical outcomes of Japanese patients with non‐muscle invasive bladder cancer receiving intravesical bacillus Calmette–Guérin treatment

**DOI:** 10.1111/iju.14933

**Published:** 2022-05-21

**Authors:** Makito Miyake, Eiji Kikuchi, Kenta Shinozaki, Yi Piao, Nobuya Hayashi, Ryo Koto, Masahisa Jinushi, Takashi Kobayashi

**Affiliations:** ^1^ Department of Urology Nara Medical University Nara Japan; ^2^ Department of Urology St. Marianna University School of Medicine Kanagawa Japan; ^3^ AstraZeneca K.K. Osaka Japan; ^4^ Department of Urology Kyoto University Graduate School of Medicine Kyoto Japan

**Keywords:** BCG vaccine, humans, Japan, treatment outcome, urinary bladder neoplasms

## Abstract

**Objectives:**

To investigate current patterns and outcomes of intravesical bacillus Calmette–Guérin treatment in Japanese patients with bladder cancer, including the proportion of patients completing induction therapy, and time to subsequent treatments.

**Methods:**

This retrospective cohort study utilized administrative claims data from the Medical Data Vision Co., Ltd. database to identify patients with a diagnosis of bladder cancer who had received ≥1 prescription of intravesical bacillus Calmette–Guérin between April 2008 and September 2015, and had ≥1 database record dated ≥12 weeks after the initial bacillus Calmette–Guérin dose. Patients were followed until September 2018, the last date of available data, or in‐hospital death. Patients receiving six doses of bacillus Calmette–Guérin at intervals of <21 days were considered to have completed induction according to guidelines. Time from initial bacillus Calmette–Guérin dose to subsequent bladder cancer treatment after the end of treatment was defined as the recurrence‐free duration.

**Results:**

Of 6140 patients identified (median age 73.0 years; 83.4% males), 4588 (74.7%) completed induction and 1552 (25.3%) did not. Median recurrence‐free duration was 64.4, 77.7, and 31.6 months in the overall, complete‐induction and incomplete‐induction cohorts, respectively. Corresponding 3‐year recurrence‐free rate was 56.3%, 59.0%, and 48.2% in these groups. The rate of cystectomy was approximately 6% at 5 years in all cohorts.

**Conclusions:**

Approximately 75% of Japanese patients who undergo intravesical bacillus Calmette–Guérin treatment receive a guideline‐compliant induction regimen, but outcomes were not satisfactory, highlighting the need for more effective treatments for non‐muscle invasive bladder cancer.

**Abbreviations & Acronyms**
BCG: bacillus Calmette–Guérin
BMI: body mass index
CI: confidence interval
CT: chemotherapy
HR: hazard ratio
IQR: interquartile range
JUA: Japanese Urological Association
MDV: Medical Data Vision Co., Ltd.
NE: non‐estimable
NMIBC: non‐muscle invasive bladder cancer
RT: radiotherapy
SD: standard deviation
TURBT: transurethral resection of bladder tumor

## INTRODUCTION

Bladder cancer is the tenth most common cancer in the world,^1^ and its incidence is projected to increase by 73% over the next 20 years.^2^ Japanese men have the highest age‐standardized incidence of bladder cancer in Central and Eastern Asia, with 9.6 cases per 100 000 diagnosed annually.^3^

Approximately 70–75% of newly diagnosed bladder cancers are classified as NMIBC.^4^, ^5^, ^6^ NMIBC can be treated with TURBT, followed by adjuvant intravesical instillation of BCG or chemotherapy. The recommended therapy is induction and maintenance therapy with intravesical BCG for intermediate‐ and/or high‐risk patients.^4^, ^5^, ^6^, ^7^ Intravesical BCG has been used for over 30 years to treat bladder cancer,^8^ and demonstrated to reduce the risk of recurrence and progression after TURBT.^9^, ^10^

The US and European guidelines recommend six once‐weekly intravesical instillations of BCG.^4^, ^6^ Although there is strong evidence to support the use of BCG for treating NIMBC, there is limited evidence to support any one regimen for BCG induction,^11^ and there are currently limited real‐world data on how BCG is administered, or what the outcomes are, during routine clinical practice in Japan. It is assumed that in the real‐world setting, various patterns of intravesical BCG treatment (including patterns not adhering to the guidelines) would be applied to patients with high‐risk NMIBC.

This study (the ‘KEGON’ study) aimed to investigate the current patterns and outcomes of intravesical BCG treatment in Japanese patients with bladder cancer, including the extent to which these adhere to guidelines, and the characteristics of patients receiving this treatment, the proportion of patients completing induction therapy with or without receiving maintenance therapy, and the treatments they received after intravesical BCG treatment has failed.

## METHODS

### Study design

This was a retrospective cohort study using secondary data from the MDV (Tokyo, Japan) anonymized administrative claims database, which includes hospital‐based claims data and Diagnosis Procedure Combination/per diem payment system data. We identified patients who had a diagnosis of bladder cancer (ICD10 code: C67x), received ≥1 prescription of intravesical BCG between April 2008 and September 2015, and had ≥1 medical record in the database dated ≥12 weeks after initial intravesical BCG. Patients were followed until September 2018, the last date of available data, or in‐hospital death, whichever came first. This study was reviewed and approved by an independent ethics committee (Non‐Profit Organization MINS Institutional Review Board, Approval ID: 200217).

The index date (Day 1) was defined as the date of the initial prescription of intravesical BCG. The BCG induction period was from the index date until Day 84 (12 weeks), and the first intravesical BCG treatment period was defined as the time from the index date to the last BCG prescription (Fig. S1).

This study included two cohorts and two sub‐cohorts as follows (Fig. S2): (i) the complete‐induction cohort included patients who received ≥6 BCG prescriptions, had intervals of <21 days between consecutive BCG prescriptions during the initial six prescriptions, and had no other treatment during the BCG induction period; (ii) the incomplete‐induction cohort included patients who did not meet the criteria for the complete‐induction cohort and whose treatment did not adhere to guidelines; (iii) the intensive‐induction subcohort included patients who met the criteria for the complete‐induction cohort and had intervals of <8 days between consecutive BCG prescriptions during the BCG induction period; and (iv) the non‐intensive‐induction subcohort included patients who met the criteria for the complete‐induction cohort but did not meet the criteria for the intensive‐induction cohort.

According to the Japanese guideline,^7^ the standard regimen is 6–8 doses of weekly intravesical BCG treatment, called “BCG induction therapy,” followed by regular intravesical BCG treatment, called “BCG maintenance therapy.” The intensive induction subcohort was defined as the guideline‐adherent group, and the incomplete induction and non‐intensive induction subcohorts as the nonadherent group.

### Endpoints

First, we described the treatment pattern of intravesical BCG and the baseline characteristics of patients in each cohort at the index date, including relevant comorbidities (renal/urinary tract cancers other than bladder cancer, genitourinary symptoms, benign prostatic hyperplasia, and autoimmune diseases). Table S1 describes how these data were derived from the database entries.

Other endpoints included the time from the index date to the beginning of the subsequent treatment for bladder cancer, which was assumed to be the date of recurrence; this parameter was therefore an estimate of recurrence‐free duration. Table S2 provides definitions of subsequent treatments. Other secondary endpoints were the time from the index date to the date of cystectomy (cystectomy‐free duration), and the subsequent treatment patterns for bladder cancer after the first intravesical BCG treatment in the total population and each cohort. The number of intravesical BCG prescriptions within the BCG induction period (from Day 1 to 84) was also assessed as an exploratory outcome.

### Statistical analysis

Continuous variables (e.g., age, duration) were summarized using means, SD, medians, minimums, maximums, and IQR as appropriate. Frequency and percentages were used to summarize categorical variables. The Kaplan–Meier method was used to assess the time to any events and to estimate median and its 95% CIs. HRs with 95% CIs between complete and incomplete inductions were estimated based on a proportional hazards model. Recurrence‐free duration and cystectomy‐free duration were calculated based on the time from the index date to the occurrence of the subsequent treatment for bladder cancer and cystectomy, respectively. All analyses were performed using SAS® Version 9.4 (SAS Institute Inc., Cary, NC, USA).

As this was a database study, the target population included patients for whom intravesical BCG was not indicated (e.g., patients with stage ≥II disease), and subsequent TURBT could be performed with diagnostic intent (rather than therapeutic), or as biopsy for suspicious lesions during the follow‐up. Therefore, sensitivity analysis was performed to examine outcomes in more limited patient populations: (i) only those patients with stage 0 or I disease at baseline; and (ii) excluding TURBT from the subsequent treatment. We also performed a sensitivity analysis in which the intensive and non‐intensive cohorts were defined by an interval of <9 or ≥9 days, respectively, between consecutive intravesical BCG prescriptions during induction to reflect the brief postponements typically encountered in clinical practice as a result of weekends and public holidays.

## RESULTS

### Patient selection in the database and BCG treatment pattern

**FIGURE 1:**
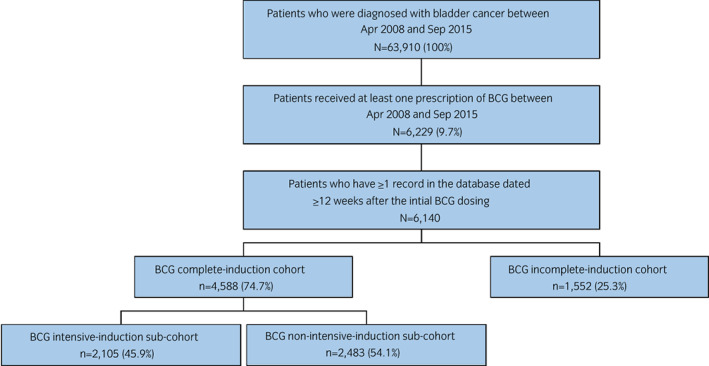
Patient disposition. [Colour figure can be viewed at wileyonlinelibrary.com]

The MDV database included data for 63 910 patients with bladder cancer between April 2008 and September 2015. Of these patients, 6140 (9.6%) met the study inclusion criteria and comprised the overall study cohort (Fig. 1); 4588 (74.7%) comprised the complete‐induction cohort and 1552 (25.3%) the incomplete‐induction cohort.

### Patient characteristics

In the overall cohort, most patients (83.4%) were male and almost two‐thirds were aged ≥70 years (63.6%; Table 1). Among 3839 patients with cancer stage data, most had stage 0 or I disease (39.2% and 52.3%, respectively), and 8.5% had stage ≥II. The most common of the comorbidities investigated were genitourinary signs and symptoms (47.3% of patients), benign prostatic hyperplasia (40.6% of men), and cancer other than bladder cancer (26.5%). Overall, 4.9% and 8.1% of patients had malignant neoplasms of the renal pelvis or ureter, respectively, in addition to bladder cancer.

**TABLE 1:** Demographic and clinical characteristics of the overall patient cohort, complete‐induction cohort and incomplete‐induction cohort

	Overall (*n* = 6140)	Complete‐induction cohort (*n* = 4588)	Incomplete‐induction cohort (*n* = 1552)
Age, years			
Mean (SD)	72.4 (9.6)	72.1 (9.5)	73.5 (9.6)
Median	73.0	73.0	75.0
Age categories, *n* (%)			
≤49 years	102 (1.7)	77 (1.7)	25 (1.6)
50–59 years	452 (7.4)	358 (7.8)	94 (6.1)
60–69 years	1683 (27.4)	1303 (28.4)	380 (24.5)
70–79 years	2375 (38.7)	1763 (38.4)	612 (39.4)
80–89 years	1439 (23.4)	1031 (22.5)	408 (26.3)
≥90 years	89 (1.4)	56 (1.3)	33 (2.1)
Sex, *n* (%)			
Male	5118 (83.4)	3875 (84.5)	1243 (80.1)
Female	1022 (16.6)	713 (15.5)	309 (19.9)
BMI, kg/m^2^	(*n* = 5154)	(*n* = 4126)	(*n* = 1028)
Mean (SD)	23.4 (3.4)	23.4 (3.3)	23.3 (3.5)
Median	23.3	23.3	23.1
Smoking history, *n* (%)	(*n* = 4824)	(*n* = 3854)	(*n* = 970)
Yes	2343 (48.6)	1893 (49.1)	450 (46.4)
No	2481 (51.4)	1961 (50.9)	520 (53.6)
Cancer stage (version 7), *n* (%)	(*n* = 3839)	(*n* = 3094)	(*n* = 745)
0	1506 (39.2)	1195 (38.6)	311 (41.7)
I	2008 (52.3)	1649 (53.3)	359 (48.2)
II	208 (5.4)	162 (5.2)	46 (6.2)
III	73 (1.9)	53 (1.7)	20 (2.7)
IV	44 (1.1)	35 (1.1)	9 (1.2)
Comorbidities, *n* (%)	(*n* = 6140)	(*n* = 4588)	(*n* = 1552)
Non‐bladder cancer	1627 (26.5)	1190 (25.9)	437 (28.2)
Renal pelvis malignancy	300 (4.9)	226 (4.9)	74 (4.8)
Ureteral malignancy	496 (8.1)	333 (7.3)	163 (10.5)
Benign prostatic hyperplasia	2079 (40.6†)	1530 (39.5†)	549 (44.2†)
Genitourinary signs/symptoms	2904 (47.3)	2242 (48.9)	662 (42.7)
Autoimmune disease	248 (4.0)	185 (4.0)	63 (4.1)
Treatments received at or before the index date, *n* (%)			
Intravesical chemotherapy	845 (13.8)	672 (14.6)	173 (11.1)
Oral corticosteroids	140 (2.3)	99 (2.2)	41 (2.6)
Dialysis	20 (0.3)	8 (0.2)	12 (0.8)
Immunosuppressive drugs	14 (0.2)	10 (0.2)	4 (0.3)
Antiplatelet drugs	542 (8.8)	402 (8.7)	140 (9.0)

† Denominator for this percentage is male patients.

Demographic and clinical characteristics of the complete‐induction cohort and the incomplete‐induction cohort are also summarized in Table 1.

### Intravesical BCG and subsequent treatment

In the overall patient group, patients received between 1 and 10 prescriptions of intravesical BCG. The median (IQR) number of prescriptions was 7.0 (6.0–8.0) in the overall group, 4.0 (3.0–6.0) in the incomplete‐induction cohort, and 8.0 (6.0–8.0) in the complete‐induction cohort (Table 2). The median (IQR) duration of follow‐up was 45.5 (35.7–59.3) months or approximately 3.74 years.

**TABLE 2:** Intravesical BCG induction treatment pattern of the overall patient cohort, complete‐induction cohort and incomplete‐induction cohort

	Overall (*n* = 6140)	Complete‐induction cohort (*n* = 4588)	Incomplete‐induction cohort (*n* = 1552)
No. of intravesical BCG prescriptions during induction therapy			
Mean (SD)	6.4 (1.8)	7.2 (0.9)	4.2 (2.0)
Median (IQR)	7.0 (6.0–8.0)	8.0 (6.0–8.0)	4.0 (3.0–6.0)
No. of prescriptions received, *n* (%)			
≤5	1147 (18.7)	0	1147 (73.9)
6–8	4964 (80.8)	4562 (99.4)	402 (25.9)
≥9	29 (0.5)	26 (0.6)	3 (0.2)
Follow‐up period, months			
Median (IQR)	45.5 (35.7–59.3)	46.0 (36.4–59.6)	43.6 (32.0–58.1)

**FIGURE 2:**
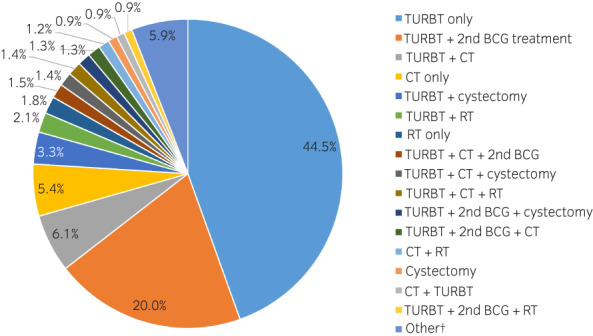
Treatments used after the first intravesical BCG treatment. †Other includes 36 individual combinations used in <20 patients (0.7%) each. [Colour figure can be viewed at wileyonlinelibrary.com]

The most common subsequent treatment after intravesical BCG was TURBT; 44.5% of patients received TURBT only, and another 20.0% of patients received TURBT and a second course of intravesical BCG (Fig. 2). All other treatments or combinations were used in <10% of patients.

### Time to subsequent treatment

**FIGURE 3:**
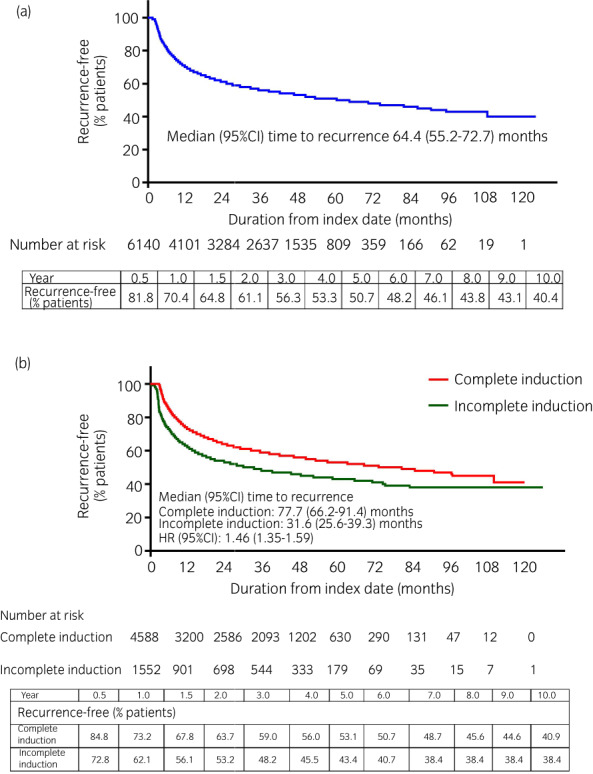
Kaplan–Meier curves of time from first intravesical BCG prescription to next subsequent treatment for bladder cancer in the (a) overall cohort and (b) complete‐ and incomplete‐induction cohorts. Recurrence was defined as the initiation of subsequent therapy for bladder cancer. [Colour figure can be viewed at wileyonlinelibrary.com]

The median duration from the index date to subsequent treatment for bladder cancer was 64.4 months (95% CI 55.2–72.7) in the overall cohort (Fig. 3a), 77.7 months (95% CI 66.2–91.4) in the complete‐induction cohort, and 31.6 months (95% CI 25.6–39.3) in the incomplete‐induction cohort (Fig. 3b).

The 6‐month, 1‐year, and 5‐year recurrence‐free rates were 81.8%, 70.4%, and 50.7%, respectively, in the overall cohort. Complete induction was associated with an increase of ~50% in the duration from initial intravesical BCG treatment to subsequent treatment compared with incomplete induction (HR 1.46; 95% CI 1.35–1.59). However, the difference was most marked in the first 3 months; thereafter, the curves were almost parallel (Fig. 3b).

Immortal time bias may impact results since patients in the complete‐induction cohort did not have early recurrence (which would have interrupted their induction treatment), whereas the patients in the incomplete cohort may have had early recurrence. The clinical characteristics of the patients with (*n* = 248) and without (*n* = 1304) early (Day 1–84) events in the incomplete‐induction cohort are provided in Table S3.

**FIGURE 4:**
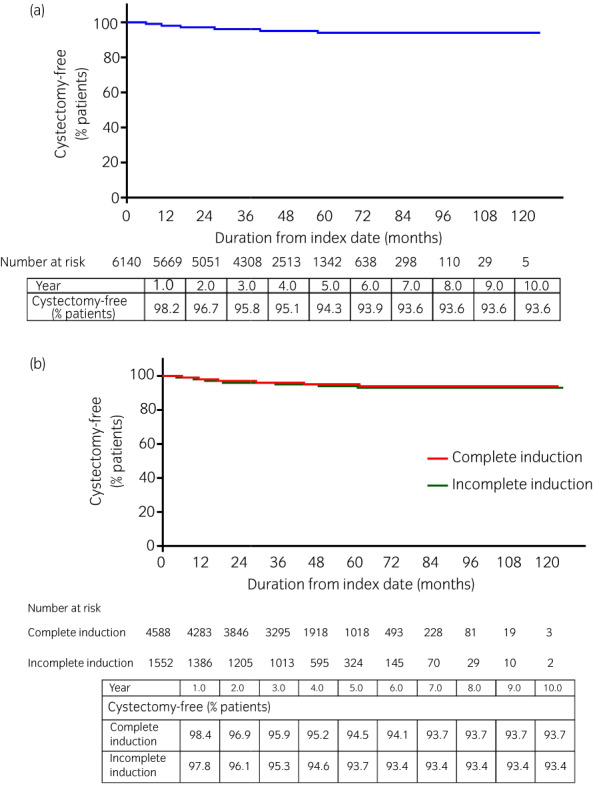
Kaplan–Meier curves of time from first intravesical BCG prescription to cystectomy in the (a) overall cohort, and (b) complete‐ and incomplete‐induction cohorts. [Colour figure can be viewed at wileyonlinelibrary.com]

The cystectomy rate after the initial intravesical BCG treatment was low in both the complete‐ and incomplete‐induction cohorts (5.5% and 6.3% at 5 years, respectively). As a result, median duration from the index date until cystectomy was not reached in any cohort (Fig. 4a,b).

The results of the sensitivity analyses were consistent with original analyses (Figs [Link], [Link]).

### Intensive induction BCG *versus* non‐intensive induction BCG

Among patients in the complete‐induction cohort, 2105 (45.9%) received intensive induction therapy with an interval of <8 days between consecutive intravesical BCG treatment, and 2483 (54.1%) received non‐intensive induction. In the latter cohort, the mode of the interval between intravesical BCG administrations was 7 days, but the maximum interval ranged from 8 to 46 days (mode of the maximum interval was 14 days). Demographic and clinical characteristics in these two cohorts are shown in Table 3.

**TABLE 3:** Demographic and clinical characteristics, and BCG induction treatment pattern, of the overall patient cohort, complete‐induction cohort, and intensive‐ and non‐intensive‐induction cohorts

	Overall (*n* = 6140)	Complete‐induction cohort (*n* = 4588)	Intensive‐induction cohort (*n* = 2105)	Non‐intensive‐induction cohort (*n* = 2483)
Age, years				
Mean (SD)	72.4 (9.6)	72.1 (9.5)	72.1 (9.4)	72.0 (9.6)
Median	73.0	73.0	73.0	73.0
Age categories, *n* (%)				
≤49 years	102 (1.7)	77 (1.7)	33 (1.6)	44 (1.8)
50–59 years	452 (7.4)	358 (7.8)	147 (7.0)	211 (8.5)
60–69 years	1683 (27.4)	1303 (28.4)	625 (29.7)	678 (27.3)
70–79 years	2375 (38.7)	1763 (38.4)	795 (37.8)	968 (39.0)
80–89 years	1439 (23.4)	1031 (22.5)	481(22.9)	550 (22.2)
≥90 years	89 (1.4)	56 (1.2)	24 (1.1)	32 (1.3)
Males, *n* (%)	5118 (83.4)	3875 (84.5)	1796 (85.3)	2079 (83.7)
BMI, kg/m^2^	(*n* = 5154)	(*n* = 4126)	(*n* = 1919)	(*n* = 2207)
Mean (SD)	23.4 (3.4)	23.4 (3.3)	23.5 (3.3)	23.4 (3.3)
Median	23.3	23.3	23.3	23.3
Smoking history, *n* (%)	(*n* = 4824)	(*n* = 3854)	(*n* = 1759)	(*n* = 2095)
Yes	2343 (48.6)	1893 (49.1)	884 (50.3)	1009 (48.2)
No	2481 (51.4)	1961 (50.9)	875 (49.7)	1086 (51.8)
Cancer stage (version 7), *n* (%)	(*n* = 3839)	(*n* = 3094)	(*n* = 1431)	(*n* = 1663)
0	1506 (39.2)	1195 (38.6)	553 (38.6)	642 (38.6)
I	2008 (52.3)	1649 (53.3)	759 (53.0)	890 (53.5)
II	208 (5.4)	162 (5.2)	79 (5.5)	83 (5.0)
III	73 (1.9)	53 (1.7)	23 (1.6)	30 (1.8)
IV	44 (1.1)	35 (1.1)	17 (1.2)	18 (1.1)

**FIGURE 5:**
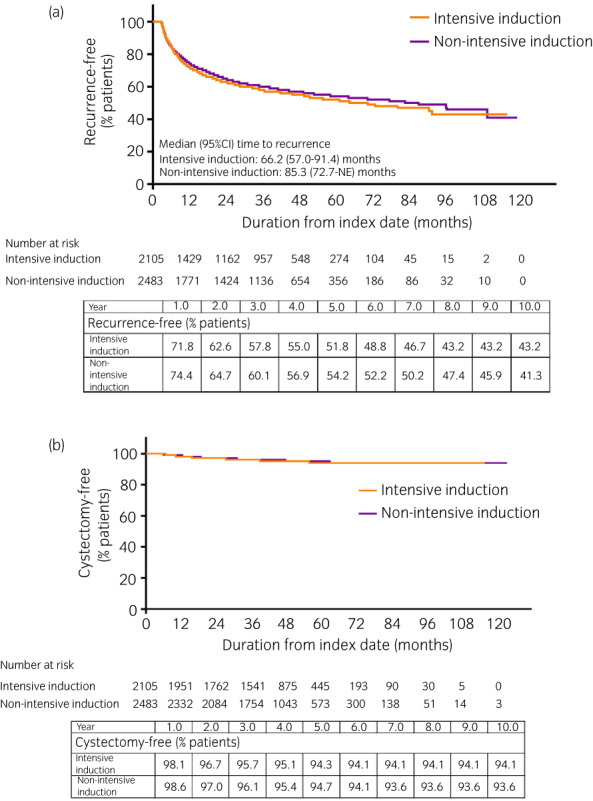
Kaplan–Meier curves of time from first intravesical BCG prescription to (a) next subsequent treatment for bladder cancer, and (b) cystectomy, in patients from the complete‐induction cohort who received intensive *versus* non‐intensive induction therapy. Recurrence was defined as the initiation of subsequent therapy for bladder cancer. [Colour figure can be viewed at wileyonlinelibrary.com]

The 6‐month, 1‐year, and 5‐year recurrence‐free rates were 84.8%, 71.8%, and 51.8%, respectively, in the intensive‐induction cohort and 84.7%, 74.4%, and 54.2%, respectively, in the non‐intensive‐induction cohort (Fig. 5a). The recurrence‐free curves (Fig. 5a) and the cystectomy‐free curves (Fig. 5b) of the two cohorts overlapped.

The results of sensitivity analyses examining the impact of a <9‐day interval between intravesical BCG pre‐scriptions were similar to the original analyses (Figs S6,S7; Table S4).

## DISCUSSION

This retrospective observational study indicates that about 75% of Japanese patients treated with intravesical BCG for bladder cancer received ≥6 intravesical doses with an interval of <21 days between consecutive doses, which we defined as complete induction therapy or “guideline‐adherent” induction therapy.

While selection bias is inherent in any retrospective database analysis, which is dependent on the quantity and quality of the data in the database, the characteristics of patients in our study can be compared with those in the previous JUA registry of patients with NMIBC (*n* = 3237).^12^ As in the JUA registry, the proportion of women with bladder cancer was <20% in our study, and our cohort included a high proportion of patients older than 70 years (63.6% *vs* 48.7% in the JUA registry).^12^ It should be noted that, unlike the JUA registry, our cohort was not limited to treatment‐naïve patients with NMIBC, which may explain our higher proportion of older patients. Based on the broad coverage of the MDV database (it is estimated that 41% of Japanese bladder cancer cases might be covered in the database), we assume that our results are generalizable to a high proportion of the Japanese population.

A 2015 consensus paper^11^ noted that no regimen has proven to be significantly more effective than the original schedule of six once‐weekly intravesical BCG injections reported by Morales *et al*. in 1976.^13^ Intravesical BCG maintenance therapy is also recommended in the guidelines; however, no standardized regimen of BCG maintenance therapy has been established, which made it difficult to accurately capture patients who received BCG maintenance therapy from the database. Therefore, we focused on intravesical BCG induction therapy in this study.

To our knowledge, this is the first study to investigate patient characteristics and outcomes in patients who completed induction therapy as described in the guidelines with those who did not complete induction, and we found that demographic and clinical characteristics were generally numerically consistent between the two cohorts.

One‐year recurrence rates were around 30% in the overall population and complete‐induction cohort. Patients who did not complete induction therapy were at higher risk of recurrence, especially early recurrence, whereas their characteristics as determined from the database were numerically consistent with those of the complete‐induction cohort. However, it is possible that some characteristics not captured from the available data and that could affect the risk of recurrence (e.g., tumor grade, multiplicity, and tumor size) were different between the two cohorts. Prior research has shown that complete response to induction BCG therapy is associated with a lower rate of recurrence.^14^ In our study, it was not possible to determine the response to induction therapy.

The 5‐year recurrence‐free rate in our study (50.7% overall and 53.1% in those who completed induction) is a little higher than the 47.0% rate reported by Malmström and colleagues in their comparison of BCG induction followed by maintenance with mitomycin C treatment.^15^ However, the 5‐year recurrence‐free rates are lower in our study than studies by Martínez‐Piñeiro *et al*., in which the rate was 62–67%,^16^ and by Farah *et al*., in which it was 69%.^17^ These differences likely reflect a number of factors including use and duration of maintenance therapy after induction, different BCG strains, and the intensity of follow‐up in clinical trials compared with routine clinical practice.

The rate of cystectomy after intravesical BCG treatment was low in our study (only approximately 6% after 5 years) and was unaffected by completion of induction therapy or by the intensity of induction therapy. The current guidelines recommend that cystectomy should be considered for patients with BCG‐unresponsive disease, according to established definitions.^18^, ^19^, ^20^, ^21^ While we were unable to identify the patterns of intravesical BCG failure (i.e., BCG unresponsiveness, BCG relapse and BCG intolerance), cystectomy was not widely performed after BCG failure in real‐world Japanese practice. In fact, the rate was lower than in a report from the US, which indicated that 29% of patients had immediate cystectomy after diagnosis of BCG‐unresponsive recurrence.^21^ Because cystectomy was so rarely performed in the current cohort of Japanese patients, our study was underpowered to detect differences in cystectomy rates between patient cohorts.

The effectiveness of intravesical BCG in NMIBC is thought to be mediated by activation of the innate immune system.^8^ Innate immunity plays an important role in the development of acquired T‐cell immunity in which immune checkpoint inhibitors (including programmed cell death protein‐1 and programmed death ligand‐1) are involved. Intravesical BCG would help effective development of T‐cell immunity and have synergy when used in combination with immuno‐oncology agents.

Since bladder cancer is an immunogenic tumor, studies are now underway to investigate the use of immune checkpoint inhibitors such as durvalumab and pembrolizumab instead of, or in combination with BCG, in patients with BCG‐naïve high‐risk NMIBC, and as treatment in patients with BCG‐unresponsive NMIBC (Table 4). In order to interpret the evidence from these clinical trials, it is necessary to establish the extent to which the comparator BCG regimens reflect current clinical use of intravesical BCG for NMIBC in the real world. Data from the current study will meet this need for Japan.

**TABLE 4:** Studies underway that investigate the use of immune checkpoint inhibitors in patients with NMIBC

NCT number (study name)	Target N	Phase	Patients	Treatment groups	Primary endpoint	Estimated date of completion
Avelumab						
NCT03892642	27	1/2	BCG‐unresponsive	Avelumab + BCG	Proportion of patients completing induction course	October 2025
NCT03950362 (PREVERT)	67	2	BCG‐unresponsive	Avelumab + radiotherapy	High‐risk recurrence‐free survival	June 2024
Durvalumab						
NCT03258593	40	1	BCG‐unresponsive	Durvalumab induction ± maintenance with vicinium induction and maintenance	Safety and tolerability	December 2022
NCT02901548	17	2	BCG‐unresponsive	Durvalumab	6‐month complete response rate	December 2021
NCT03759496	39	2	BCG‐unresponsive	Durvalumab	Maximum tolerated dose Possibility of rate of high‐grade relapse‐free after start of durvalumab 1‐year high‐grade relapse‐free rate	December 2021
NCT03317158 (ADAPT‐BLADDER)	186	1/2	BCG‐unresponsive	Durvalumab monotherapy (phase 1) Durvalumab + BCG (phase 2) Durvalumab + radiotherapy (phase 2)	Recommended dose (phase 1) 6‐month relapse‐free survival (phase 2)	March 2023
NCT04106115	64	1b/2	BCG‐unresponsive	Durvalumab + 5‐peptide cancer vaccine (S‐488210/S‐488211)	Dose‐limiting toxicity (phase 1) 1‐year disease‐free survival (phase 2)	July 2027
NCT03528694 (POTOMAC)	1018	3	BCG‐naïve	Durvalumab + BCG induction and maintenance Durvalumab + BCG induction only BCG induction and maintenance	Disease‐free survival	November 2024
Pembrolizumab						
NCT02808143	9	1	BCG‐unresponsive	Pembrolizumab preinduction then BCG induction and maintenance	Maximum tolerated dose	February 2022
NCT02324582	13	1	BCG‐unresponsive	Pembrolizumab induction + BCG induction	Safety	December 2022
NCT03504163	37	2	BCG‐naive	Pembrolizumab	6‐month disease‐free rate	April 2022
NCT04387461	37	2	BCG‐unresponsive	Pembrolizumab + CG0070	Complete response rate	June 2022
NCT04164082	161	2	BCG‐unresponsive	Pembrolizumab + gemcitabine	6‐month complete response rate 18‐month event‐free survival	March 2023
NCT02625961 (KEYNOTE‐057)	260	2	BCG‐unresponsive	Pembrolizumab	Complete response rate Disease‐free survival rate	July 2023
NCT03711032 (KEYNOTE‐676)	1525	3	BCG‐naïve or BCG‐unresponsive NMIBC	BCG induction and pembrolizumab + BCG maintenance (full or reduced maintenance regimen in BCG‐naïve cohort) BCG induction and maintenance	Complete response rate Event‐free survival	November 2024
Sasanlimab						
NCT04165317 (CREST)	999	3	BCG‐naive	Sasanlimab + BCG induction and maintenance Sasanlimab + BCG induction only BCG induction and maintenance	Event‐free survival	December 2026

Because this study used the MDV database, there are several limitations. First, the database does not merge data from an individual patient receiving treatment at more than one hospital, so if a patient had received any treatment for bladder cancer at another hospital, these data would not be identified. Therefore, patients who transferred to other healthcare facilities during the course of their treatment were lost to follow‐up. Second, the MDV database does not record deaths that occur outside hospital; therefore we were not able to evaluate survival in this study. Third, the first BCG prescription captured in the database might not be the first one for the patient, and subsequent treatments might not be fully captured. As a result, the recurrence‐free duration rate might be overestimated. Furthermore, the database does not provide information on the indication for BCG. As such, we were not able to identify patients for whom BCG was used to treat carcinoma *in situ*, or those who received BCG as maintenance therapy. Since maintenance use of BCG is recognized as having an influence on tumor recurrence, this may have affected our results, particularly in the complete‐induction cohort. Similarly, we were not able to differentiate between TURBT use for diagnosis or for treatment. Future prospective studies are expected to address these by collecting all relevant clinical information for more comprehensive analysis. Finally, the database included data for all patients, irrespective of whether they were treated according to guidelines; this explains why our analysis included some patients with stage II–IV bladder cancer in whom BCG is not indicated. It is possible that patients with an initial classification of stage II–IV disease received BCG after having undergone bladder preservation therapy (e.g., TURBT, radiation) to treat stage 0–I residual or recurrent disease, but we are unable to confirm this from the available data. We did not exclude the patients with stage II–IV disease because this would have contravened the study objective of describing the real‐world use of BCG in patients with bladder cancer.

In conclusion, in Japan, 74.7% of bladder cancer patients who start induction treatment with intravesical BCG completed it. Completion of such treatment is associated with less recurrence; thus, completion is important even if the intervals between each dose are prolonged due to adverse events. Approximately 30% of patients need subsequent treatment within a year of starting intravesical BCG treatment for NIMBC, and 44% need subsequent treatment within 3 years, indicating that more effective treatments are needed for NIMBC.

## AUTHOR CONTRIBUTIONS

Makito Miyake: Conceptualization; methodology; writing – review and editing. Eiji Kikuchi: Conceptualization; methodology; writing – review and editing. Kenta Shinozaki: Conceptualization; methodology; writing – original draft; writing – review and editing. Yi Piao: Data curation; methodology; writing – review and editing. Nobuya Hayashi: Formal analysis; methodology; writing – review and editing. Ryo Koto: Data curation; methodology; writing – review and editing. Masahisa Jinushi: Conceptualization; funding acquisition; methodology; writing – review and editing. Takashi Kobayashi: Conceptualization; methodology; writing – review and editing.

## CONFLICT OF INTEREST

EK has received honoraria from NIPPON KAYAKU and AstraZeneca K.K. KS, YP, NH, RK, and MJ are employees of AstraZeneca K.K. and hold stocks of AstraZeneca PLC. MJ was an employee of AstraZeneca K.K. at the time this analysis was conducted. TK has received research support from AstraZeneca K.K.

## APPROVAL OF THE RESEARCH PROTOCOL BY AN INSTITUTIONAL REVIEWER BOARD

This study was reviewed and approved by an independent ethics committee (Non‐profit Organization MINS Institutional Review Board, Approval ID: 200217).

## INFORMED CONSENT

N/A.

## REGISTRY AND THE REGISTRATION NO. OF THE STUDY/TRIAL

N/A.

## ANIMAL STUDIES

N/A.

## Supporting information


**Figure S1.** Schematic diagram of periods used to define the cohorts and outcomes.
**Figure S2.** Schematic diagram of the definitions of each cohort and subcohort.

**Figure S3.** Kaplan–Meier curve of time from first intravesical BCG prescription to next subsequent treatment for bladder cancer among patients with stage 0 or 1 disease (overall cohort).

**Figure S4.** Kaplan–Meier curve of time from first intravesical BCG prescription to cystectomy among patients with stage 0 or 1 disease (overall cohort).

**Figure S5.** Kaplan–Meier curve of time from first intravesical BCG administration to next subsequent treatment other than TURBT in (a) the overall cohort, and (b) the complete‐ and incomplete‐induction cohorts.

**Figure S6.** Kaplan–Meier curve of time from first intravesical BCG prescription to next subsequent treatment for bladder cancer in the newly defined intensive (i.e. <9‐day interval) and non‐intensive (i.e. ≥9‐day interval) BCG induction sub‐cohort.

**Figure S7.** Kaplan–Meier curve of time from first intravesical BCG prescription to cystectomy in the newly defined intensive (i.e. <9‐day interval) and non‐intensive (i.e. ≥9‐day interval) BCG induction sub‐cohort.

**Table S1.** Definitions use to derive patient characteristics from the database entries.
**Table S2.** Definitions for bladder cancer treatments.
**Table S3.** Demographic and clinical characteristics of patients in the incomplete‐induction cohort who did or did not experience an early event.
**Table S4.** Demographic and clinical characteristics of patients in the complete‐induction cohort who received intensive *versus* non‐intensive induction, based on a definition of intensive induction where the interval between consecutive BCG prescriptions was <9 days.
