# Supplementary material for: Real‐world treatment patterns and clinical outcomes of Japanese patients with non‐muscle invasive bladder cancer receiving intravesical bacillus Calmette–Guérin treatment
Source: Int J Urol. 2022 May 21;29(10):1120–9. doi: 10.1111/iju.14933 (PMC9790662; doi:10.1111/iju.14933)

# Supporting information

## Figures

**Fig. S1** Schematic diagram of the periods used to define the cohorts and outcomes. BCG, Bacillus Calmette–Guérin.

BCG induction period: From Day 1 to Day 84.

BCG induction therapy: Consecutive intravesical BCG prescriptions from the initial BCG prescription during BCG induction period with less than 21 days of intervals between each prescription.

1st BCG treatment: Consecutive intravesical BCG prescriptions from the initial intravesical BCG prescription during study period to the last intravesical BCG prescription prior to the beginning of the 1st subsequent treatment or the date of last available record in the database whichever comes first


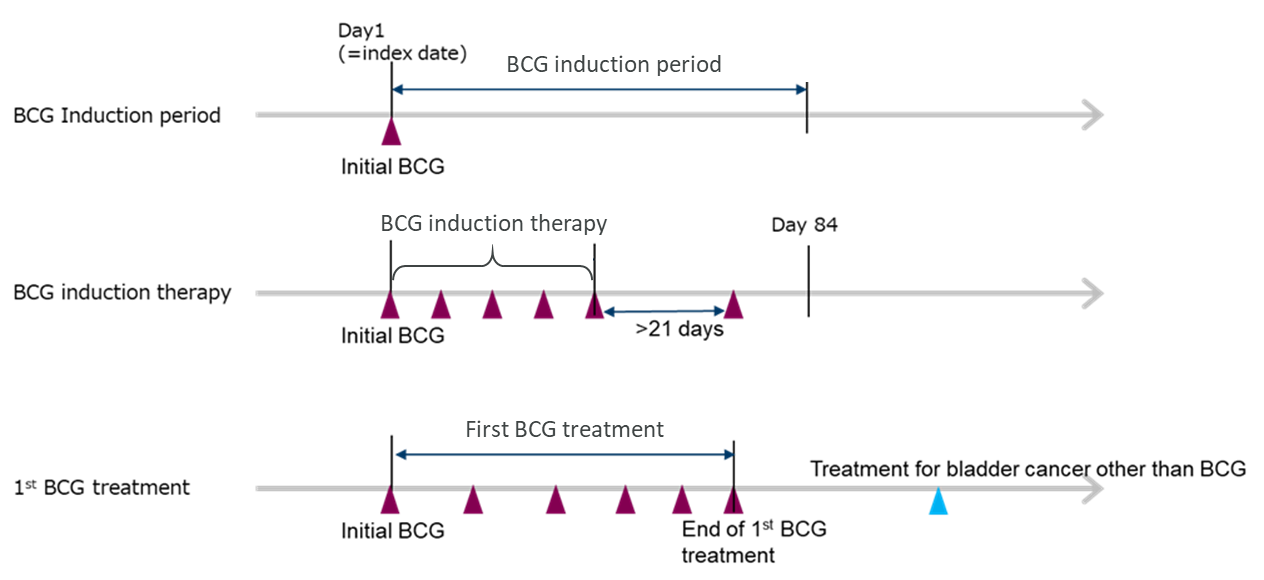


**Fig. S2** Schematic diagram of the definitions of each cohort and subcohort. BCG, Bacillus Calmette–Guérin; ICD, International Statistical Classification of Diseases and Related Health Problems


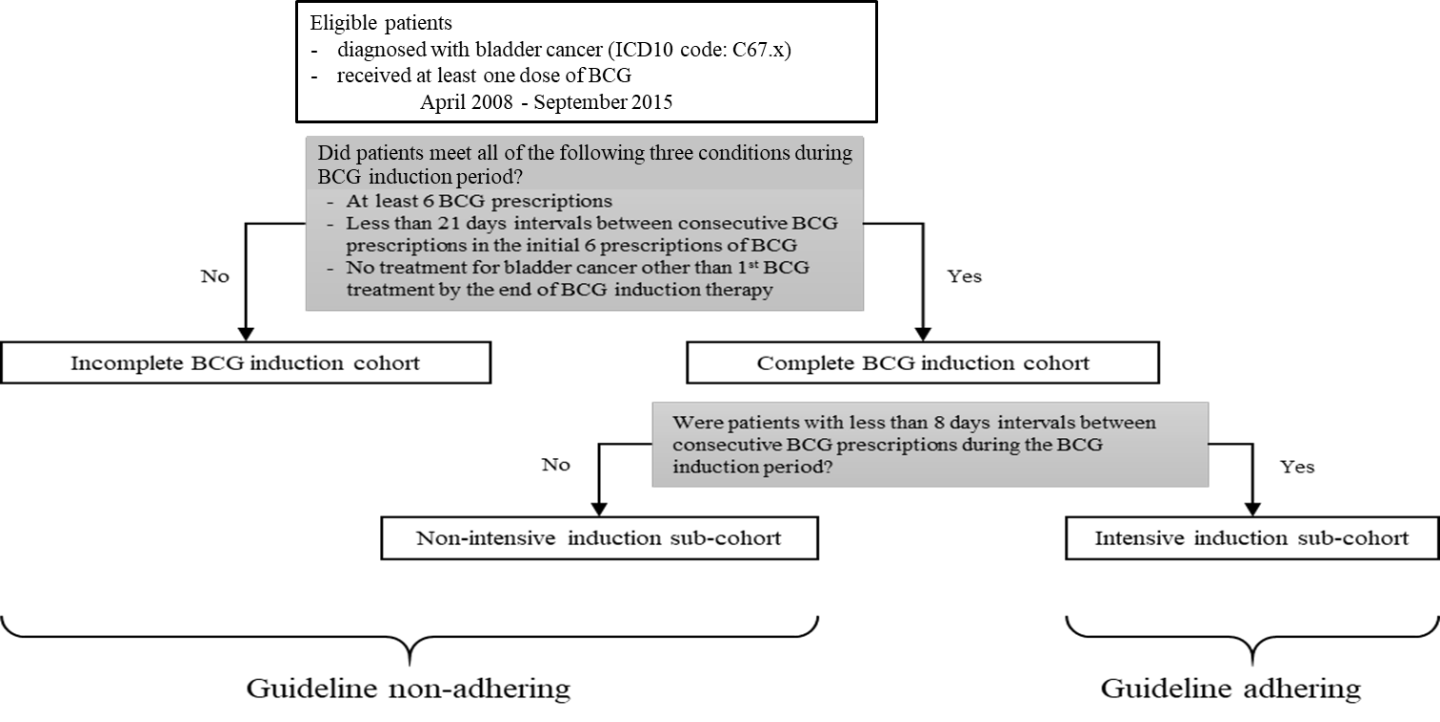

Supplement: Supplementary file 1 — Figure S1. Schematic diagram of periods used to define the cohorts and outcomes. Figure S2. Schematic diagram of the definitions of each cohort and subcohort. [file IJU-29-1120-s001.docx]
