# Supplementary figures and images for: Real‐world treatment patterns and clinical outcomes of Japanese patients with non‐muscle invasive bladder cancer receiving intravesical bacillus Calmette–Guérin treatment
Source: Int J Urol. 2022 May 21;29(10):1120–9. doi: 10.1111/iju.14933 (PMC9790662; doi:10.1111/iju.14933)

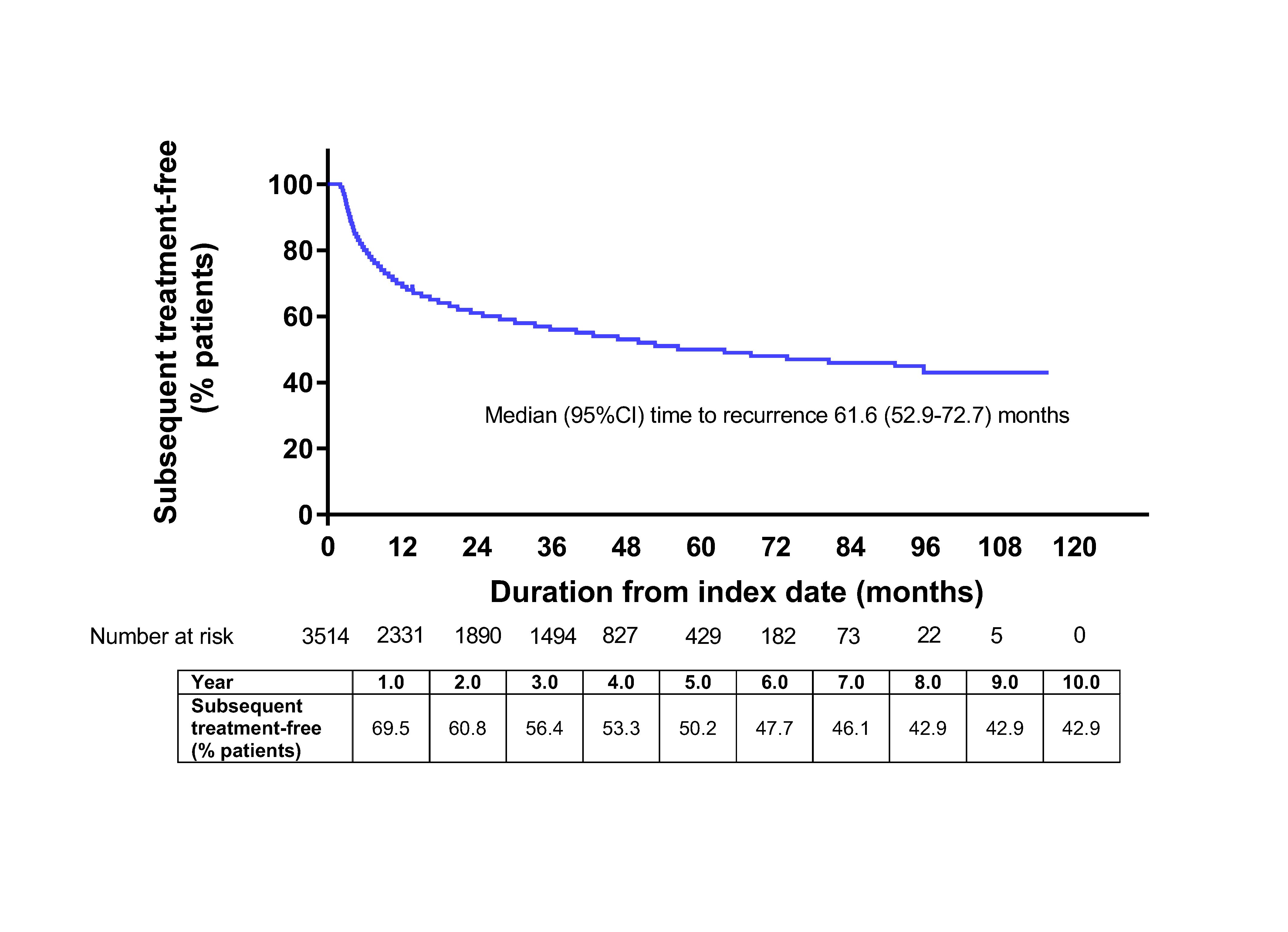

Supplement: Supplementary file 2 — Figure S3. Kaplan–Meier curve of time from first intravesical BCG prescription to next subsequent treatment for bladder cancer among patients with stage 0 or 1 disease (overall cohort). [file IJU-29-1120-s005.jpg]

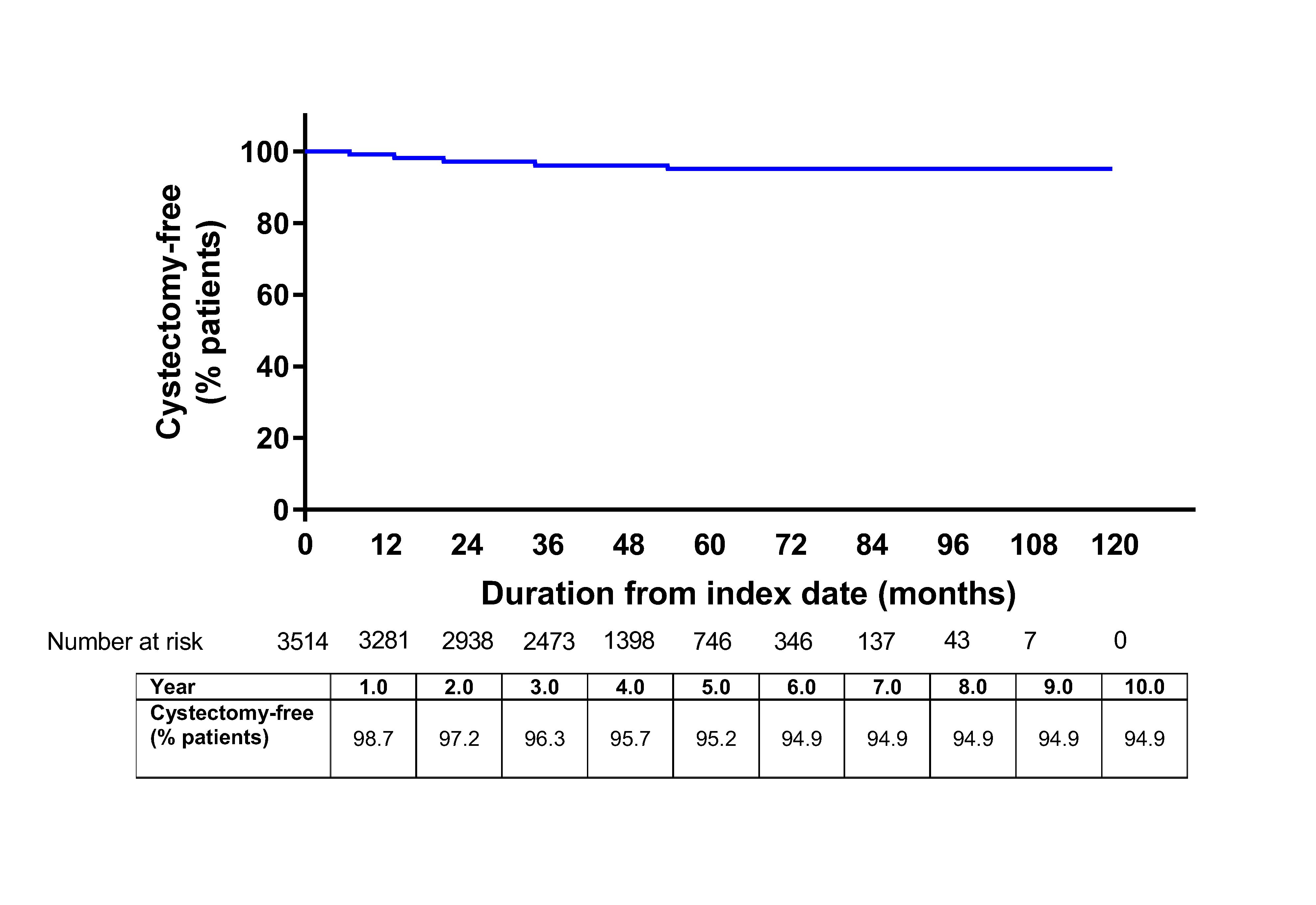

Supplement: Supplementary file 3 — Figure S4. Kaplan–Meier curve of time from first intravesical BCG prescription to cystectomy among patients with stage 0 or 1 disease (overall cohort). [file IJU-29-1120-s007.jpg]

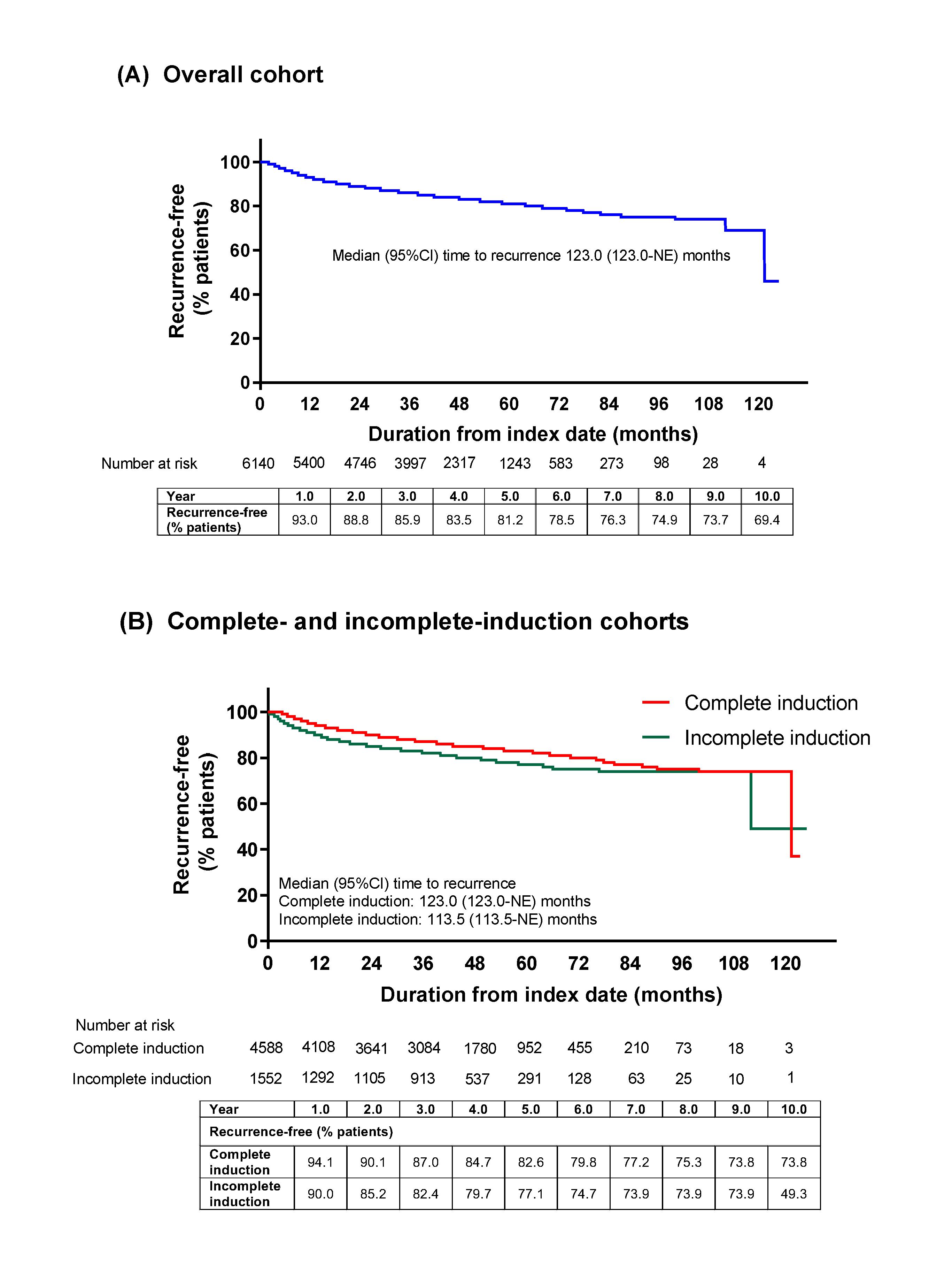

Supplement: Supplementary file 4 — Figure S5. Kaplan–Meier curve of time from first intravesical BCG administration to next subsequent treatment other than TURBT in (a) the overall cohort, and (b) the complete‐ and incomplete‐induction cohorts. [file IJU-29-1120-s004.jpg]

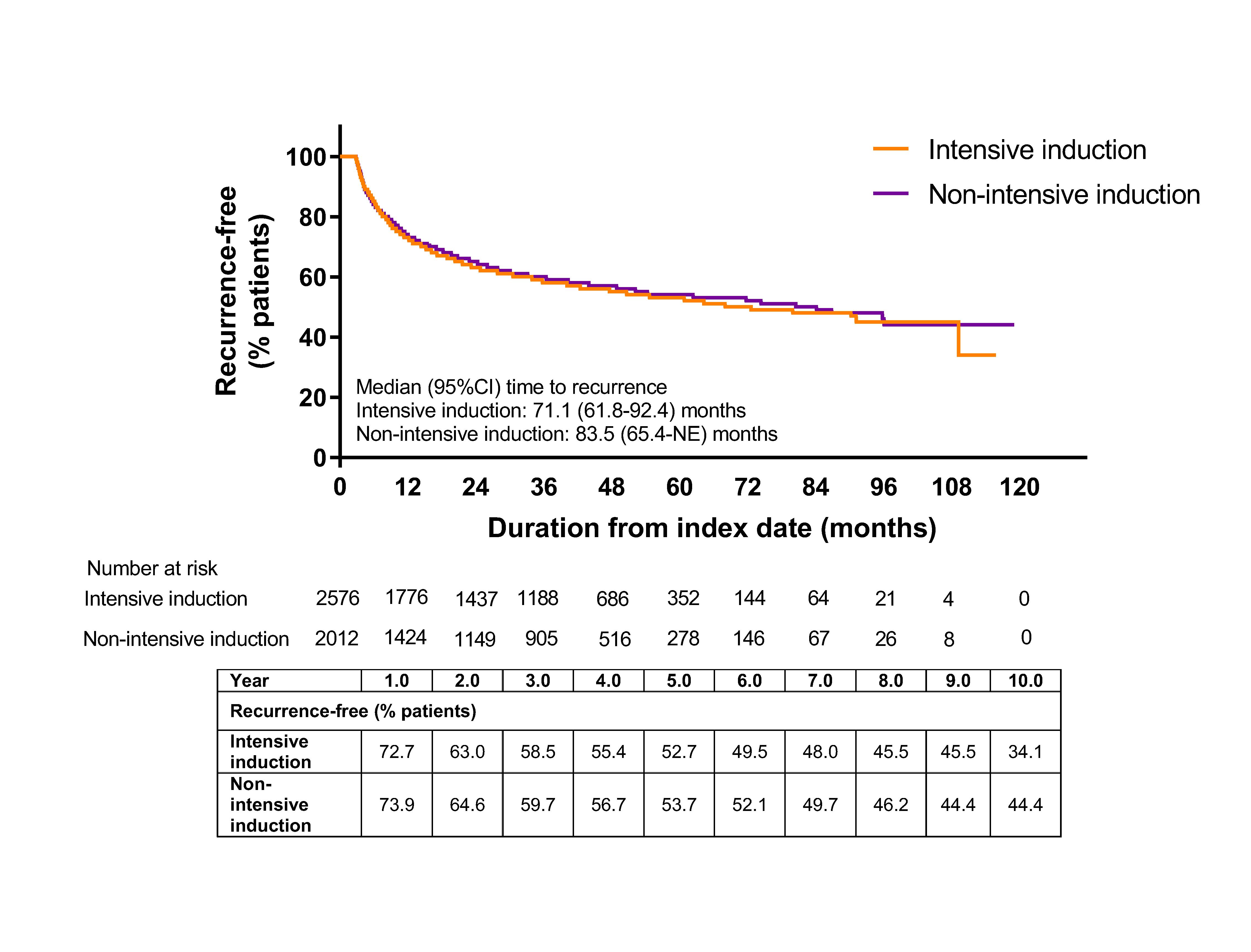

Supplement: Supplementary file 5 — Figure S6. Kaplan–Meier curve of time from first intravesical BCG prescription to next subsequent treatment for bladder cancer in the newly defined intensive (i.e. <9‐day interval) and non‐intensive (i.e. ≥9‐day interval) BCG induction sub‐cohort. [file IJU-29-1120-s003.jpg]

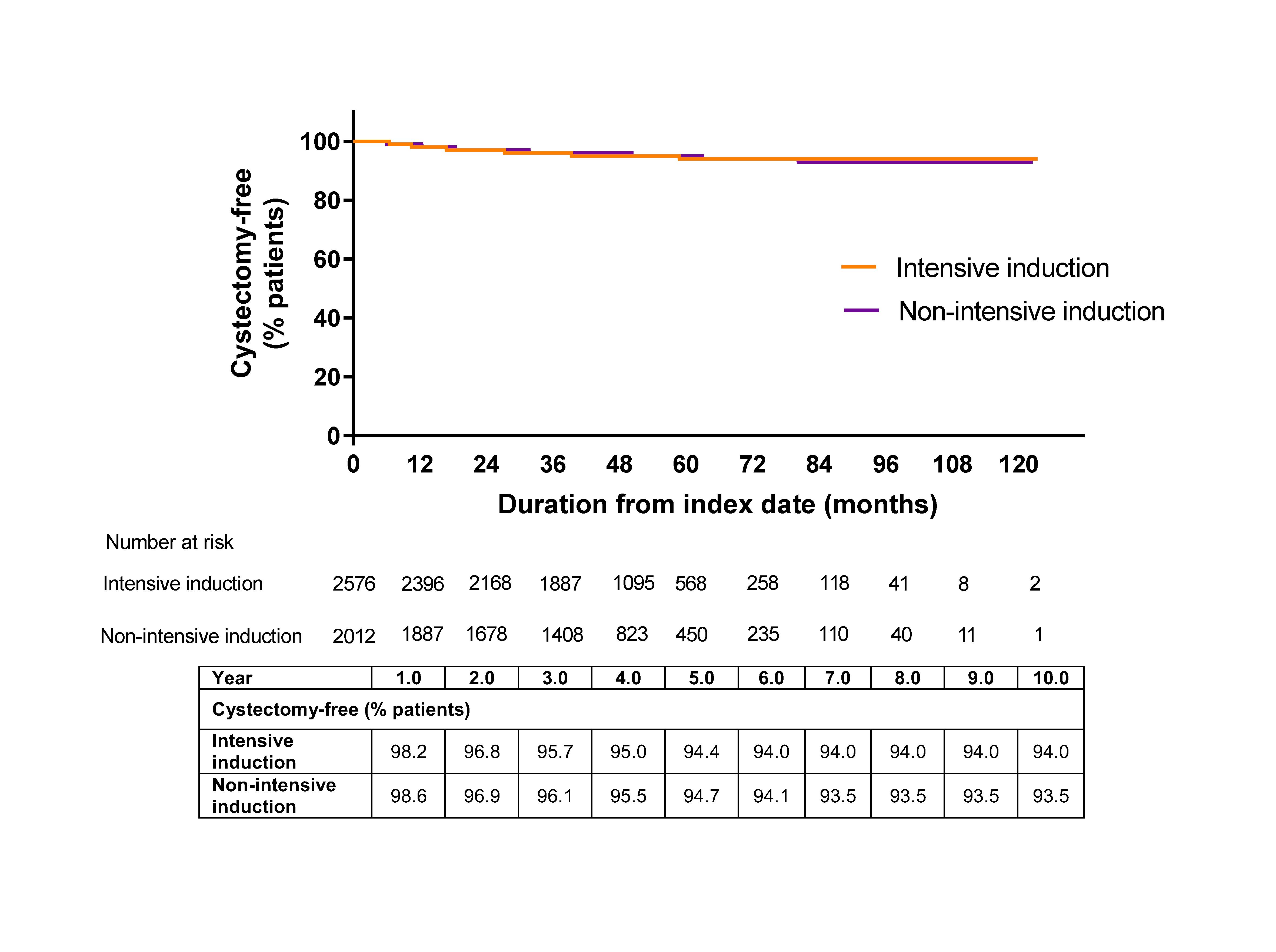

Supplement: Supplementary file 6 — Figure S7. Kaplan–Meier curve of time from first intravesical BCG prescription to cystectomy in the newly defined intensive (i.e. <9‐day interval) and non‐intensive (i.e. ≥9‐day interval) BCG induction sub‐cohort. [file IJU-29-1120-s002.jpg]
