# Supplementary material for: Real‐world treatment patterns and clinical outcomes of Japanese patients with non‐muscle invasive bladder cancer receiving intravesical bacillus Calmette–Guérin treatment
Source: Int J Urol. 2022 May 21;29(10):1120–9. doi: 10.1111/iju.14933 (PMC9790662; doi:10.1111/iju.14933)
Supplement: Supplementary file 7 — Table S1. Definitions use to derive patient characteristics from the database entries. Table S2. Definitions for bladder cancer treatments. Table S3. Demographic and clinical characteristics of patients in the incomplete‐induction cohort who did or did not experience an early event. Table S4. Demographic and clinical characteristics of patients in the complete‐induction cohort who received intensive versus non‐intensive induction, based on a definition of intensive induction where the interval between consecutive BCG prescriptions was <9 days. [file IJU-29-1120-s006.docx]

**Tables**

**Table S1** Definitions use to derive patient characteristics from the database entries

| Characteristic | | Definition |
| --- | --- | --- |
| Demographics | Age | Age at index date |
|  | Sex | Sex |
|  | BMI | “weight” and “height” recorded in discharge summary during hospitalization corresponding to the index date or before (the record closest to the index date was used).  BMI was calculated as follows:  “weight” / “height”/ ” height” * 10000 |
|  | Smoking history | As recorded in the discharge summary closest to the index date |
|  | Stage information | As recorded in the discharge summary closest to the index date |
| Comorbidity | Cancer other than bladder cancer | ICD 10 codes C00 to C97, excluding C67, at the index date or before |
|  | Heart failure | ICD 10 codes I50, I11.0, I13.0, I13.2 , at the index date or before |
|  | Type 2 diabetes | ICD 10 codes E11 or E14, at the index date or before |
|  | Benign prostatic hyperplasia | ICD 10 code N40, at the index date or before |
|  | Genitourinary signs or symptoms | ICD 10 codes R30 to R39, at the index date or before |
|  | Autoimmune diseases | ICD 10 codes D51.0, D59.1, D69.3, E05.0, E06.3, E10, E27.1, G35, G61.0, H20, K50-K51,K73, K74.3, K90.0, L10, L12, L40 (excluding L40.4), L63, L80.9, M05-M06, M08, M31.3, M33, M31.5-M31,6, M35.3, G70.0, M34, M32.1, M32.9, M35.0, M45.9, at the index date or before |
| Treatment history | Intravesical chemotherapy | Mitomycin C (ATC code: L01DC03), doxorubicin (ATC code: L01DB01), epirubicin (ATC code: L01DB03), pirarubicin (ATC code: L01DB08) or gemcitabine (ATC code: L01BC05) at the index date or before |
|  | Oral corticosteroids | ATC code H02 at the index date or before |
|  | Dialysis | Procedure code J038 at the index date or before |
|  | Immunosuppressive drugs | ATC code L04 at the index date or before |
|  | Antiplatelet drugs | ATC code B01Ac at the index date or before |

ATC, Anatomical Therapeutic Chemical; BMI, body mass index; ICD, International Classification of Diseases.

**Table S2** Definitions for bladder cancer treatments

| Treatment for bladder cancer | Definition |
| --- | --- |
| First INTRAVESICAL BCG treatment | Consecutive BCG prescriptions (ATC code: L03A9) from the first BCG prescription during the study period to the last BCG prescription prior to the beginning of the first subsequent treatment or the date of last available record in the database, whichever comes first |
| Transurethral resection of the bladder tumor (TURBT) | TURBT (Procedure code: K803-6) was classified into two categories:   1. TURBT only: without following BCG prescription(s) after the index date 2. TURBT with second BCG treatment: TURBT with following BCG prescriptions after the index date |
| Second INTRAVESICAL BCG treatment | BCG prescription(s) following TURBT after the index date |
| Systemic therapy | Systemic therapy for bladder cancer included the following medications:   - - Methotrexate (ATC code: L01BA01)   - Pembrolizumab (ATC code: L01XC18) - Paclitaxel (ATC code: L01CD01) - Nedaplatin (ATC code: L01XA01) - Docetaxel (ATC code: L01CD02) - Doxorubicin (ATC code: L01DB01) - Cisplatin (ATC code: L01XA01) - Cyclophosphamide (ATC code: L01AA01)   - Gemcitabine (ATC code: L01BC05)   - Carboplatin (ATC code: L01XA02)   - Epirubicin (ATC code: L01DB03)   - Ifosfamide (ATC code: L01AA06)   - Vinblastine (ATC code: L01CA01)   Systemic therapy other than pembrolizumab was defined as chemotherapy, and classified as chemotherapy without cystectomy or chemotherapy with cystectomy (see definition of cystectomy) |
| Cystectomy | Cystectomy (Procedure code: K803-00) will be classified to following categories:   1. Cystectomy with neoadjuvant chemotherapy only: Cystectomy with chemotherapy administered within 90 days before cystectomy 2. Cystectomy with adjuvant chemotherapy only: Cystectomy with chemotherapy administered within 90 days after cystectomy 3. Cystectomy only: Cystectomy without neoadjuvant and adjuvant chemotherapy 4. Cystectomy with neoadjuvant chemotherapy and adjuvant chemotherapy: Cystectomy with chemotherapy administered within 90 days before and after cystectomy |
| Radiotherapy | Procedure code M001 |

ATC, Anatomical Therapeutic Chemical; BCG, Bacillus Calmette–Guérin; TURBT, transurethral resection of the bladder tumor.

**Table S3** Demographic and clinical characteristics of patients in the incomplete-induction cohort who did or not experience an early event, i.e., an event in the first 1–84 days after the first prescription of intravesical BCG

|  | Incomplete-induction cohort | |
| --- | --- | --- |
|  | Any event between day 1 and 84 (n=248) | No event between day 1 and 84 (n=1,304) |
| Age, years |  |  |
| Mean (SD) | 71.4 (9.5) | 73.8 (9.6) |
| Median (Q1–Q3) | 73.0 (65.0–78.0) | 75.0 (67.0–81.0) |
| Age categories, n (%) |  |  |
| ≤49 years | 7 (2.8) | 18 (1.4) |
| 50–59 years | 16 (6.5) | 78 (6.0) |
| 60–69 years | 75 (30.2) | 305 (23.4) |
| 70–79 years | 101 (40.7) | 511 (39.2) |
| 80–89 years | 47 (19.0) | 361 (27.7) |
| ≥90 years | 2 (0.8) | 31 (2.4) |
| Sex, n (%) |  |  |
| Male | 192 (77.4) | 1051 (80.6) |
| Female | 56 (22.6) | 253 (19.4) |
| BMI, kg/m^2^ | (n=192) | (n=836) |
| Mean (SD) | 23.6 (3.4) | 23.2 (3.5) |
| Median (Q1–Q3) | 23.6 (21.0–25.5) | 23.1 (20.7–25.3) |
| Smoking history, n (%) | (n=192) | (n=778) |
| Yes | 95 (49.5) | 355 (45.6) |
| No | 97 (50.5) | 423 (54.4) |
| Cancer stage (version 8), n (%) | (n=167) | (n=578) |
| 0 | 63 (37.7) | 248 (42.9) |
| I | 80 (47.9) | 279 (48.3) |
| II | 12 (7.2) | 34 (5.9) |
| III | 10 (6.0) | 16 (2.8) |
| IV | 2 (1.2) | 1 (0.2) |
| Comorbidities, n (%) | (n=248) | (n=1304) |
| Non-bladder cancer | 77 (31.0) | 360 (27.6) |
| Heart failure | 18 (7.3) | 112 (8.6) |
| Type 2 diabetes | 56 (22.6) | 320 (24.5) |
| Benign prostatic hyperplasia | 80 (32.3) | 469 (36.0) |
| Genitourinary signs/symptoms | 115 (46.4) | 547 (41.9) |
| Autoimmune disease | 15 (6.0) | 48 (3.7) |
| Treatments received at or before the index date, n (%) |  |  |
| Intravesical chemotherapy | 31 (12.5) | 142 (10.9) |
| Oral corticosteroids | 11 (4.4) | 30 (2.3) |
| Dialysis | 1 (0.4) | 11 (0.8) |
| Immunosuppressive drugs | 3 (1.2) | 1 (0.1) |
| Antiplatelet drugs | 24 (9.7) | 116 (8.9) |

BMI, body mass index; Q1 or Q3, quartile 1 or 3; SD, standard deviation.

**Table S4** Demographic and clinical characteristics of patients in the complete-induction cohort who received intensive versus non-intensive induction, based on a definition of intensive induction where the interval between consecutive BCG prescriptions was <9 days

|  | Overall (n=6140) | Complete-induction cohort (n=4588) | Intensive-induction cohort (n=2576) | Non-intensive-induction cohort (n=2012) |
| --- | --- | --- | --- | --- |
| Age, years |  |  |  |  |
| Mean (SD) | 72.4 (9.6) | 72.1 (9.5) | 72.0 (9.5) | 72.1 (9.5) |
| Median (Q1–Q3) | 73.0 (66.0–79.0) | 73.0 (66.0–79.0) | 73.0 (66.0–79.0) | 73.0 (66.0–79.0) |
| Age categories, n (%) |  |  |  |  |
| ≤49 years | 102 (1.7) | 77 (1.7) | 44 (1.7) | 33 (1.6) |
| 50–59 years | 452 (7.4) | 358 (7.8) | 193 (7.5) | 165 (8.2) |
| 60–69 years | 1,683 (27.4) | 1,303 (28.4) | 757 (29.3) | 548 (27.2) |
| 70–79 years | 2,375 (38.7) | 1,763 (38.4) | 970 (37.6) | 798 (39.6) |
| 80–89 years | 1,439 (23.4) | 1,031 (22.5) | 590 (22.8) | 444 (22.0) |
| ≥90 years | 89 (1.4) | 56 (1.3) | 29 (1.1) | 27 (1.3) |
| Males, n (%) | 5,118 (83.4) | 3,875 (84.5) | 2,203 (85.3) | 1,678 (83.3) |
| BMI, kg/m^2^ | (n=5,154) | (n=4,126) | (n=1,922) | (n=2,212) |
| Mean (SD) | 23.4 (3.4) | 23.4 (3.3) | 23.5 (3.4) | 23.4 (3.3) |
| Median | 23.3 | 23.3 | 23.3 | 23.3 |
| Smoking history, n (%) | (n=4,824) | (n=3,854) | (n=2,166) | (n=1,688) |
| Yes | 2,343 (48.6) | 1,893 (49.1) | 1,086 (50.1) | 807 (47.8) |
| No | 2,481 (51.4) | 1,961 (50.9) | 1,080 (49.9) | 881 (52.2) |
| Cancer stage (version 8), n (%) | (n=3,839) | (n=3,094) | (n=1,750) | (n=1,344) |
| 0 | 1,506 (39.2) | 1,195 (38.6) | 675 (38.6) | 520 (38.7) |
| I | 2,008 (52.3) | 1,649 (53.3) | 930 (53.1) | 719 (53.5) |
| II | 208 (5.4) | 162 (5.2) | 86 (4.9) | 76 (5.7) |
| III | 107 (2.8) | 81 (2.6) | 54 (3.1) | 27 (2.0) |
| IV | 10 (0.3) | 7 (0.2) | 5 (0.3) | 2 (0.1) |
| Cancer stage (version 7), n (%) | (n=3,839) | (n=3,094) | (n=1,750) | (n=1,344) |
| 0 | 1,506 (39.2) | 1,195 (38.6) | 675 (38.6) | 520 (38.7) |
| I | 2,008 (52.3) | 1,649 (53.3) | 930 (53.1) | 719 (53.5) |
| II | 208 (5.4) | 162 (5.2) | 86 (4.9) | 76 (5.7) |
| III | 73 (1.9) | 53 (1.7) | 35 (2.0) | 18 (1.3) |
| IV | 44 (1.1) | 35 (1.1) | 24 (1.4) | 11 (0.8) |
| eGFR, n (%) | (n=690) | (n=575) | (n=353) | (n=222) |
| ≥60 mL/min/1.73m^2^ | 461 (66.8) | 393 (68.3) | 247 (70.0) | 146 (65.8) |
| <60 mL/min/1.73m^2^ | 229 (33.2) | 182 (31.7) | 106 (30.0) | 76 (34.2) |

BCG, Bacillus Calmette–Guérin; BMI, body mass index; eGFR, estimated glomerular filtration rate; Q1 or Q3, quartile 1 or 3; SD, standard deviation.
